# 1,2-Dichloroethane Induces Neuronal Apoptosis Through AMPK-mTOR-ULK1 Pathway Activation and Autophagic Flux Impairment

**DOI:** 10.3390/biology15181580

**Published:** 2026-09-09

**Authors:** Zhenlin Luo, Chen Wang, Chunting Wang, Gaoyang Wang, Wanting Cui, Zijiang Wang, Fenghong Zhao

**Affiliations:** 1 Department of Occupational and Environmental Health, School of Public Health, China Medical University, Shenyang 110122, China; 2 Department of Clinical Genetics, Shengjing Hospital of China Medical University, Shenyang 110004, China; 3 Liaoning Provincial Center for Disease Control and Prevention, Shenyang 110172, China; 4 Key Laboratory of Environmental Stress and Chronic Disease Control and Prevention, Ministry of Education, China Medical University, Shenyang 110122, China

**Keywords:** 1,2-dichloroethane, 2-chloroethanol, AMPK-mTOR-ULK1, autophagy, apoptosis

## Abstract

Prolonged exposure to 1,2-dichloroethane (1,2-DCE) can lead to cognitive dysfunction, and neuronal loss is a proven key driver. In mice, 1,2-DCE exposure reduced hippocampal ATP levels, triggering AMPK phosphorylation, mTOR suppression, and ULK1 activation, along with increased Beclin 1 and LC3-II/I levels, collectively indicating enhanced autophagy initiation. Meanwhile, p62 accumulation and decreased LAMP1 and CTSD expression indicated impaired autophagic degradation, accompanied by increased neuronal apoptosis. Consistent with these in vivo findings, PC12 cells, a neuron-like cell line, exhibited mitochondrial dysfunction after exposure to 2-chloroethanol (2-CE, a metabolite of 1,2-DCE in vivo), as evidenced by ATP depletion, mitochondrial membrane potential loss, and elevated ROS production. Furthermore, 2-CE activated the AMPK-mTOR-ULK1 signaling pathway to initiate autophagy, while also disrupting lysosomal structure and function, leading to impaired autophagic flux and enhanced apoptosis. In conclusion, prolonged 1,2-DCE exposure induces mitochondrial dysfunction and ATP depletion, activating AMPK-mTOR-ULK1-mediated autophagy initiation. Meanwhile, concomitant lysosomal damage impairs autophagic degradation, and the combined effect promotes neuronal apoptosis. These findings suggest a potential strategy for preventing and treating 1,2-DCE-induced cognitive impairment.

## Simple Summary

1,2-Dichloroethane (1,2-DCE) is a widely used industrial solvent known to cause brain damage and cognitive dysfunction. However, the precise mechanisms underlying this neurotoxicity remain unclear. This study demonstrates that prolonged exposure to 1,2-DCE disrupts energy production in neurons, activating the AMPK-mTOR-ULK1 signaling pathway, which triggers the cellular self-cleaning process known as autophagy. At the same time, 1,2-DCE damages the lysosomes, the cellular structures responsible for waste degradation, thereby impairing autophagic flux. This combination of excessive autophagy activation and impaired degradation ultimately leads to neuronal cell death. Our findings reveal a novel dual mechanism underlying 1,2-DCE-induced cognitive impairment and identify the AMPK-mTOR-ULK1 pathway as a potential therapeutic target for the prevention and treatment of this condition.

## 1. Introduction

1,2-Dichloroethane (1,2-DCE) is widely applied in industry and agriculture. It is a key raw material for manufacturing vinyl chloride monomers, and also serves as a grain fumigant and soil sterilant [1]. 1,2-DCE enters the human body via respiratory inhalation, gastrointestinal ingestion and dermal contact, then rapidly distributes to the systemic circulation and peripheral tissues, among which the brain is the primary target organ [2]. Metabolized by cytochrome P450 2E1 (CYP2E1), 1,2-DCE is converted into 2-chloroethanol (2-CE), a highly reactive and more toxic intermediate that induces brain tissue injury [2]. Epidemiological studies have consistently demonstrated that long-term 1,2-DCE exposure causes neurological symptoms including dizziness, insomnia and memory decline in humans [3,4]. Consistently, our prior studies have demonstrated that 1,2-DCE exposure triggers anxiety-like behaviors and cognitive impairment in mice [5,6]. Collectively, these observations establish that 1,2-DCE induces central nervous system (CNS) neurotoxicity, yet the underlying molecular mechanisms remain poorly elucidated and warrant further in-depth investigation.

Autophagy is a core molecular pathway for the preservation of cellular and organismal homeostasis, exerting cytoprotective effects through multiple pathways, including the removal of damaged mitochondria, degradation of apoptotic effector proteins, scavenging of reactive oxygen species, provision of metabolic energy, and relief of endoplasmic reticulum stress [7]. It is a dynamic, multi-step process, termed autophagic flux, that encompasses autophagosome formation, maturation, fusion with lysosomes, and subsequent degradation of engulfed cargo by lysosomal hydrolases [8]. Several core proteins serve as essential indicators of autophagic activity. LC3 is lipidated to LC3-II and incorporated into the autophagosome membrane, serving as a specific marker of autophagosome formation. p62/SQSTM1 binds to LC3 and is selectively degraded by autophagy. Its accumulation therefore indicates impaired autophagic degradation. Beclin 1 is a core component of the class III PI3K complex that initiates autophagosome formation. Dysregulation of this process can manifest in two distinct forms: insufficient autophagy [9] and excessive autophagic activation [10]. Either excessive autophagy beyond a certain threshold or reduced autophagy flux will compromise cell survival [11]. Dysregulated autophagy encompasses any deviation from normal autophagic homeostasis, which may include insufficient autophagic flux, excessive activation, or both [12]. Autophagy dysfunction is linked to the pathogenesis of major human disorders [13]. In the nervous system, neurons are post-mitotic cells with no proliferative capacity. Precise homeostatic control of autophagy is critical for neuronal survival and physiological function [14].To date, the exact role of autophagy in 1,2-DCE-triggered neurotoxicity remains largely uncharacterized.

Multiple intracellular signaling pathways modulate autophagy, among which the AMPK-mTOR-ULK1 axis is the most canonical and centrally regulatory [15]. AMP-activated protein kinase (AMPK) acts as a cellular energy sensor activated by multiple stressors, with energy deprivation as the primary stimulus [16]. Under energy-replete conditions, elevated intracellular ATP levels bind to the γ-regulatory subunit of AMPK, inhibiting its kinase activity. Conversely, AMP accumulation during energy deprivation potently activates AMPK. Mammalian target of rapamycin (mTOR) and Unc-51-like kinase 1 (ULK1) are two pivotal downstream effectors of AMPK in autophagy initiation [17,18]. Activated AMPK initiates autophagy through a dual regulatory mechanism: it phosphorylates and inhibits mTOR to relieve mTOR-mediated repression of ULK1, and directly phosphorylates ULK1 for its activation. Our previous work has verified that 1,2-DCE exposure reduces ATP content in mouse brain tissue. Nevertheless, the regulatory role of the AMPK-mTOR-ULK1 pathway in autophagy activation during 1,2-DCE neurotoxicity remains undefined.

Accumulating evidence has revealed that 1,2-DCE exposure induces apoptosis of cerebellar granule cells (CGCs) and cerebral cortical neurons in mice, as well as human CGCs in vitro [16,19]. Building on these prior observations, the present study aimed to test the hypothesis that prolonged 1,2-DCE exposure activates the AMPK-mTOR-ULK1 signaling cascade, subsequently inducing dysregulated neuronal autophagy and subsequent cell death. Our findings will provide novel mechanistic insights into the molecular basis of 1,2-DCE-induced neurotoxicity.

## 2. Materials and Methods

### 2.1. Animals

Six-week-old healthy female Kunming mice (24.0 ± 2.0 g) were obtained from the Laboratory Animal Center of China Medical University. All mice were housed in a standard animal facility under controlled conditions: temperature, 22 ± 2 °C; relative humidity, 50 ± 10%; and a 12 h light/dark cycle. After a 7-day acclimation period, mice (n = 15 per group) were exposed to 0, 0.225, 0.45, and 0.9 g/m^3^ of 1,2-DCE (≥99.0%; Sinopharm, Ningbo, China) in 100-L static inhalation chambers, with 5 mice per chamber during each exposure session. The 1,2-DCE was weighed according to the target concentration, placed on a plate suspended in the chamber, and rapidly evaporated by a built-in fan after sealing. Due to the chamber capacity, the 15 mice in each group were exposed in three sequential batches (5 mice per batch) daily, with freshly generated 1,2-DCE vapor for each session. The daily exposure duration was 3.5 h for 8 consecutive weeks. Our previous study has verified the stability of the 1,2-dichloroethane concentration in the static inhalation chamber, with oxygen concentration close to 20%, carbon dioxide below 2%, and humidity below 70% at the end of exposure [1]. Mice had ad libitum access to food and drinking water except during exposure periods. Body weight and general behavioral status were recorded throughout the experiment.

After 8 weeks of exposure, cognitive function was evaluated using the novel object recognition (NOR) test and the Morris water maze (MWM) test according to a previously described protocol [6]. In the NOR test, the recognition index (RI) during the training phase was calculated as (exploration time of a single familiar object/total exploration time of the two familiar objects A and B) × 100%. In the test phase, RI was defined as (exploration time of familiar object A or novel object C/total exploration time of objects A and C) × 100%. The discrimination index (DI) was calculated as [(exploration time of novel object C − exploration time of familiar object A)/total exploration time of objects A and C] × 100%. In the MWM test, escape latency during the acquisition phase and residence time in the target quadrant during the probe trial were analyzed. Subsequently, mice were euthanized by cervical dislocation under CO_2_ anesthesia, and hippocampal tissues were rapidly isolated for subsequent experiments. All animal experimental procedures were approved by the Animal Ethics Committee of China Medical University and performed in strict accordance with institutional animal care guidelines.

### 2.2. Cell Culture and Treatment

Differentiated PC12 cells were purchased from the Cell Bank of the Chinese Academy of Medical Sciences (TCR9, Shanghai, China). Cells were cultured in DMEM medium (01-052-1ACS, BioInd, Kibbutz Beit Haemek, Israel) supplemented with 10% fetal bovine serum and 1% penicillin–streptomycin mixture (03-031-1B, BioInd, Israel), and maintained at 37 °C in a humidified incubator containing 5% CO_2_. PC12 cells were treated with 0, 22.5, 45, and 90 mM 2-CE (>99.0%, Sinopharm, Ningbo, China), a metabolite of 1,2-DCE in vivo, for 24 h. For pharmacological intervention, cells were pretreated with various agents for 1 h prior to 2-CE (90 mM) exposure: autophagy activator rapamycin (Rapa; GC15031, GlpBio, Montclair, CA, USA; 100 nM), autophagy inhibitor 3-methyladenine (3-MA; GC10710, GlpBio, Montclair, CA, USA; 1 mM), chloroquine (CQ; GC19549, GlpBio, Montclair, CA, USA; 10 μM), AMPK inhibitor Compound C (CC; GC17243, GlpBio, Montclair, CA, USA; 0.5 μM), and trehalose (G8570, Sinopharm, Ningbo, China; 100 mM).

### 2.3. Transmission Electron Microscopy (TEM)

Fresh hippocampal tissues were cut into 1 mm^3^ pieces and immediately immersed in ice-cold glutaraldehyde phosphate buffer, followed by post-fixation with osmium tetroxide (OsO_4_) for 2 h. Samples were dehydrated with a gradient ethanol solution, embedded in epoxy resin mixed with acetone, and cut into ultrathin sections using an ultramicrotome (Leica UC7, Leica, Wetzlar, Germany). The sections were stained with uranyl acetate and lead citrate. Neuronal ultrastructure in the hippocampus was observed and imaged under a transmission electron microscope (Hitachi 7650, Tokyo, Japan) at 10,000× magnification.

### 2.4. Detection of ATP Content

Intracellular ATP levels were measured using an ATP Assay Kit (S0026, Beyotime, Shanghai, China) according to the manufacturer’s instructions. Briefly, hippocampal tissues or PC12 cells were lysed with ATP lysis buffer, and protein concentrations were determined using a BCA Protein Assay Kit (P0012, Beyotime, China). Equal volumes of sample lysate and ATP detection reagent were mixed, and luminescence intensity was measured using a microplate reader (Synergy H1, BioTek, Winooski, VT, USA). ATP content was normalized to the corresponding protein concentration.

### 2.5. Observation of Cell Morphology

PC12 cells were seeded into 6-well plates at a density of 1 × 10^5^ cells/well. After 24 h of 2-CE treatment, cell morphology was observed and photographed under a bright-field microscope (BX51, Olympus, Tokyo, Japan).

### 2.6. Detection of Cell Viability

Cell viability was determined using a Cell Counting Kit-8 (CCK-8; GK10001, GlpBio, Montclair, CA, USA). PC12 cells were seeded into 96-well plates at a density of 5 × 10^3^ cells/well. After 24 h of treatment, 10 μL CCK-8 reagent was added to each well containing 100 μL medium, and plates were incubated at 37 °C for 2 h. Absorbance at 450 nm was measured using a microplate reader (Synergy H1, BioTek, USA).

### 2.7. Detection of ROS Levels

Intracellular ROS levels were measured using a Reactive Oxygen Species Assay Kit (E-BC-K138-F, Elabscience, Wuhan, China). PC12 cells were incubated with 10 μM DCFH-DA at 37 °C for 40 min in the dark. Cells were then harvested by centrifugation at 1000 rpm for 10 min at 4 °C and resuspended in PBS. Fluorescence intensity was measured using a fluorescence microplate reader (Synergy H1, BioTek, USA) with excitation and emission wavelengths of 488 nm and 525 nm, respectively.

### 2.8. Detection of Mitochondrial Membrane Potential (MMP)

MMP was detected using a JC-1 assay kit (E-CK-A301, Elabscience, China). PC12 cells were incubated with JC-1 working solution at 37 °C for 20 min in the dark. After washing twice with 1× JC-1 assay buffer, cells were observed and photographed under an inverted fluorescence microscope (IX70, Olympus, Japan).

### 2.9. Ad-RFP-GFP-LC3B Transfection

PC12 cells were seeded into 12-well plates and transfected with RFP-GFP-LC3B adenovirus at a multiplicity of infection (MOI) of 200, then incubated for 24 h. After transfection, cells were treated with fresh medium containing 90 mM 2-CE for another 24 h. The positive control group was pretreated with 100 nM Rapa for 1 h followed by routine culture for 24 h. For the inhibitor group, cells were pretreated with CC for 1 h, then exposed to 90 mM 2-CE for 24 h. Autophagic flux was observed under a laser scanning confocal microscope (FV1000S-IX81, Olympus, Japan).

### 2.10. Western Blot Assay

Hippocampal tissues or PC12 cells were lysed with RIPA lysis buffer (P0013B, Beyotime, China). Total protein concentration was determined by the BCA method. The primary antibodies used in this study were as follows: Bax (1:1000, A20227, ABclonal, Wuhan, China), Bcl-2 (1:1000, A19693, ABclonal), cleaved caspase 3 (1:1000, YM343, Immunoway, San Jose, CA, USA), LC3B (1:1000, A19665, ABclonal), Beclin 1 (1:1000, A21191, ABclonal), p62 (1:1000, WL02385, Wanleibio, Shenyang, China), lysosome-associated membrane protein 1 (LAMP1, 1:1000, A23947, ABclonal), cathepsin D (CTSD, 1:1000, 74089, Cell Signaling Technology, Danvers, MA, USA), p-AMPK (Thr172, 1:1000, WL05103, Wanleibio), AMPK (1:1000, WL02254, Wanleibio), p-mTOR (Ser2448, 1:1000, AP0115, ABclonal), mTOR (1:1000, WL02477, Wanleibio), p-ULK1 (Ser555, 1:1000, YP1542, Immunoway), ULK1 (1:1000, WL03067, Wanleibio), GAPDH (1:2000, sc-32233, Santa Cruz, Dallas, TX, USA), and β-actin (1:2000, sc-47778, Santa Cruz). Protein bands were visualized using an ECL chemiluminescence kit (WLA006a, Wanleibio, China). After chemiluminescence exposure, band images were collected and quantitatively analyzed using ImageJ software version 1.53e.

### 2.11. Statistical Analysis

Data are presented as means ± SD from 3–5 mice per group (for in vivo studies) or from three independent experiments (for in vitro studies). Statistical analysis was performed using GraphPad Prism 9.0 software. One-way and two-way analysis of variance (ANOVA) followed by SNK post hoc test was used for multiple group comparisons. A *p* value < 0.05 was considered statistically significant.

## 3. Results

### 3.1. 1,2-DCE Exposure Induces Cognitive Dysfunction and Hippocampal Neuronal Apoptosis in Mice

**Figure 1:**
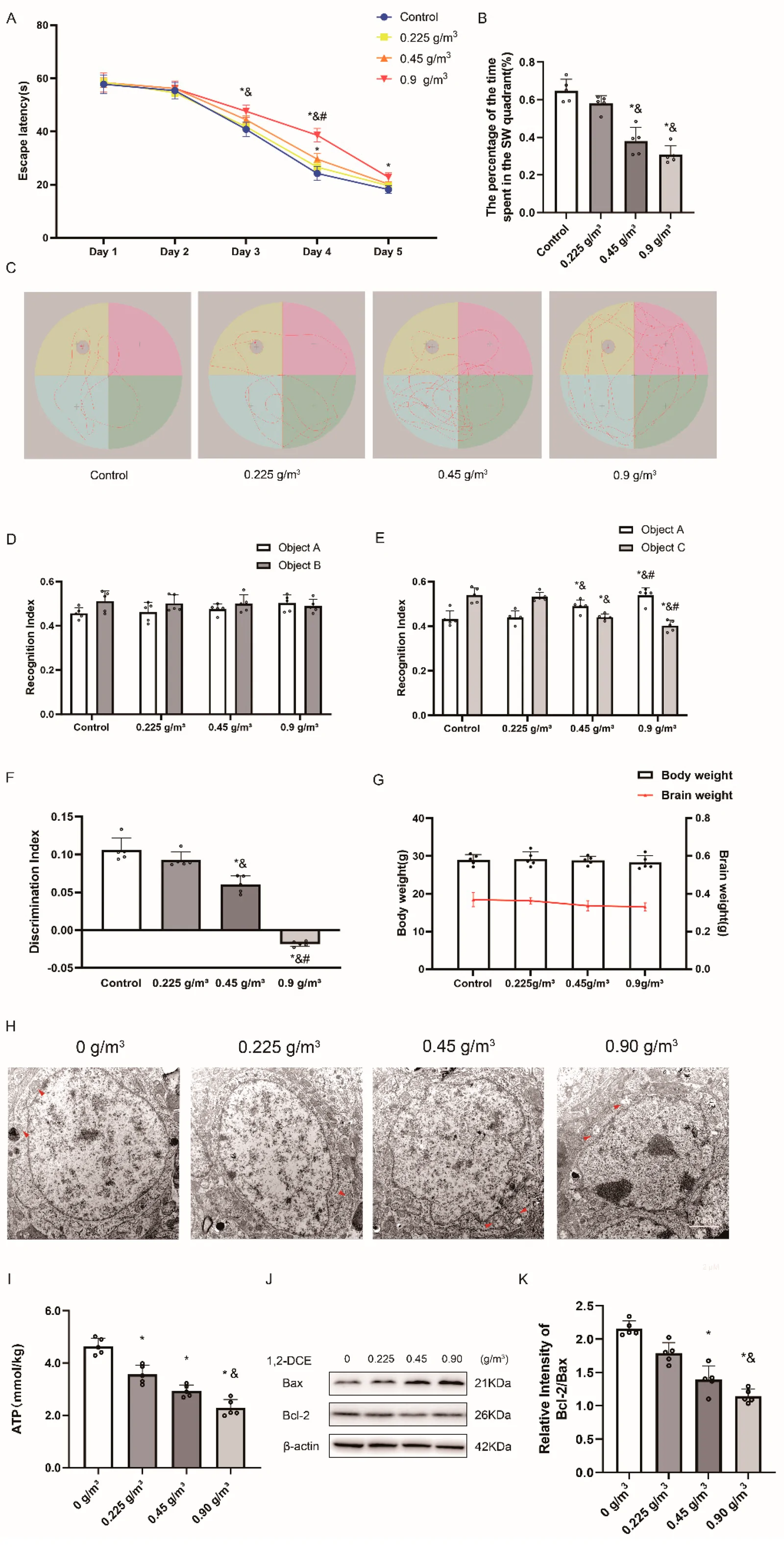
1,2-DCE exposure induces cognitive impairment and hippocampal neuronal apoptosis in mice. (**A**) Escape latency during MWM training. n = 5. (**B**) Proportion of time spent in the target quadrant during the probe trial. n = 5. (**C**) Representative trajectory (red lines) images from the probe test. (**D**) RI in the training phase. n = 5. (**E**) RI in the test phase. n = 5. (**F**) DI in the test phase. n = 5. (**G**) Body weight and brain weight of mice. n = 5. (**H**) Ultrastructure of hippocampal CA1 neurons. n = 3. Scale bar: 2 µm; mitochondria (red arrows). (**I**) ATP levels in hippocampal tissues. n = 5. (**J**) Representative western blot bands of BAX and Bcl-2. (**K**) Quantitative analysis of the Bcl-2/Bax ratio. n = 5. *, vs. control; &, vs. 0.225 g/m^3^; #, vs. 0.45 g/m^3^, *p* < 0.05.

During the MWM training phase, escape latency was significantly prolonged in mice exposed to 0.9 g/m^3^ of 1,2-DCE compared with the control group from days 3 to 5 (*p* < 0.05, Figure 1A). In the probe trial, residence time in the target quadrant was reduced by 41% and 52% in the 0.45 and 0.9 g/m^3^ groups, respectively (*p* < 0.05, Figure 1B). The trajectories of 1,2-DCE-exposed mice were more scattered and disorderly relative to those of control mice (Figure 1C). In the novel object recognition test, no significant difference in RI was observed among all groups during the training phase (Figure 1D). In the test phase, however, mice exposed to 0.45 and 0.9 g/m^3^ 1,2-DCE exhibited significant reductions in RI and DI for novel object C (*p* < 0.05, Figure 1E,F). No significant changes in body weight or brain weight were detected after 1,2-DCE exposure (Figure 1G).

TEM revealed varying degrees of ultrastructural damage in hippocampal CA1 neurons following 1,2-DCE treatment, including blurred cell membrane integrity, irregular nuclear morphology, and mitochondrial swelling (Figure 1H). Hippocampal ATP levels were decreased by 22%, 35% and 50% in the 0.225 (*p* = 0.0424), 0.45 (*p* = 0.003) and 0.9 g/m^3^ (*p* = 0.0003) groups, respectively (Figure 1I). The Bcl-2/Bax ratio was markedly downregulated in the 0.45 and 0.9 g/m^3^ groups relative to the control (*p* < 0.0001, Figure 1J,K).

### 3.2. 1,2-DCE Exposure Activates the AMPK-mTOR-ULK1 Pathway and Impairs Autophagic Flux in the Mouse Hippocampus

**Figure 2:**
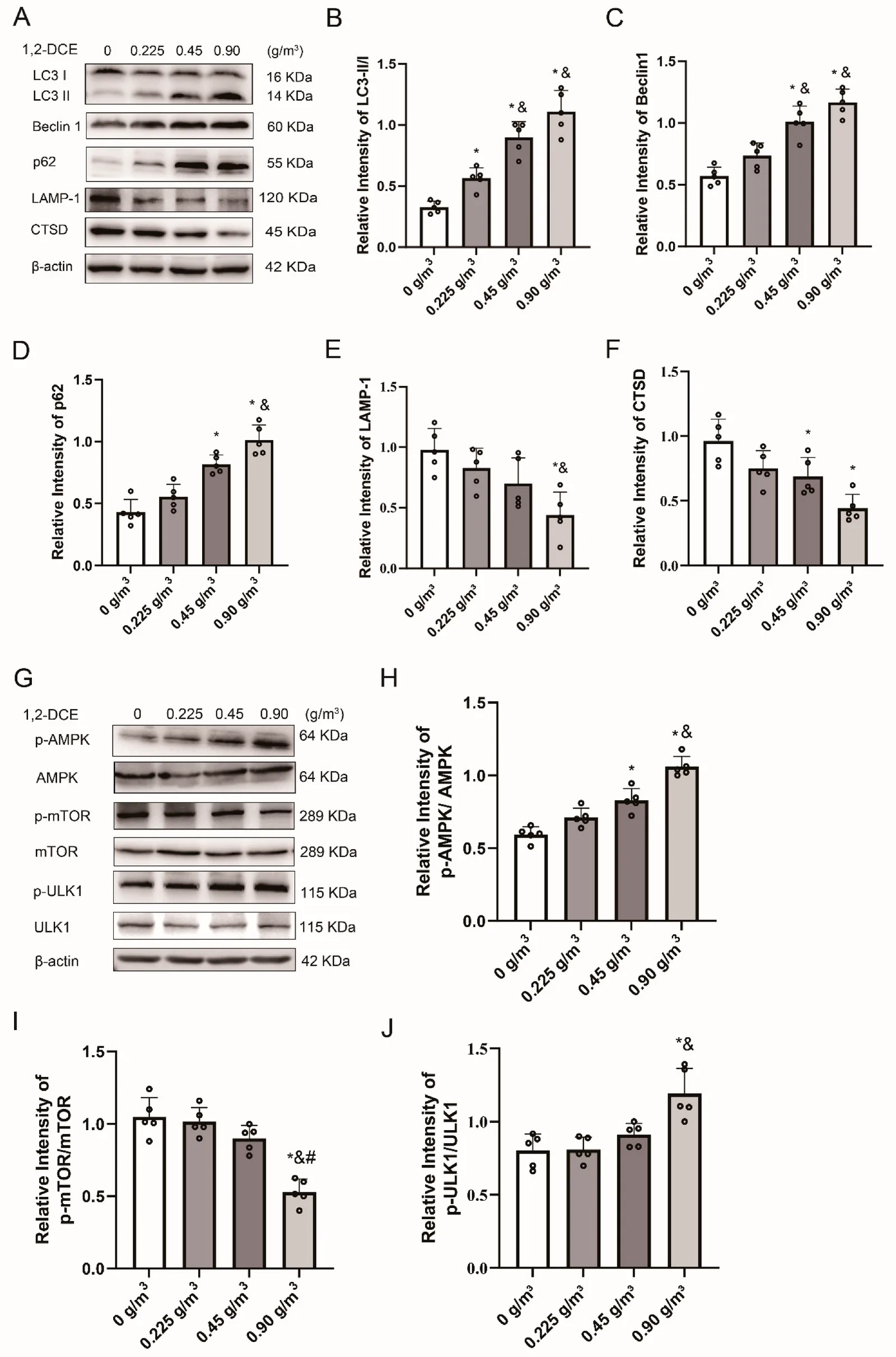
1,2-DCE exposure activates the AMPK-mTOR-ULK1 pathway and impairs autophagic flux in the mouse hippocampus. (**A**) Representative western blot bands of LC3-II/I, Beclin 1, p62, LAMP1 and CTSD. (**B**) Quantitative analysis of LC3-II/I expression. (**C**) Quantitative analysis of Beclin 1 expression. (**D**) Quantitative analysis of p62 expression. (**E**) Quantitative analysis of LAMP1 expression. (**F**) Quantitative analysis of CTSD expression. (**G**) Representative western blot bands of p-AMPK, AMPK, p-mTOR, mTOR, p-ULK1 and ULK1. (**H**) Quantitative analysis of the p-AMPK/AMPK ratio. (**I**) Quantitative analysis of the p-mTOR/mTOR ratio. (**J**) Quantitative analysis of the p-ULK1/ULK1 ratio. n = 5. *, vs. control; &, vs. 0.225 g/m^3^; #, vs. 0.45 g/m^3^, *p* < 0.05.

The protein expression levels of LC3-II/I, Beclin 1 and p62 were significantly elevated in the 0.45 and 0.9 g/m^3^ groups compared with the control (*p* < 0.0001, Figure 2A–D). By contrast, the expression of the lysosomal marker LAMP1 (*p* = 0.0017) was markedly inhibited in the 0.9 g/m^3^ group and CTSD (*p* = 0.0337, *p* = 0.0001) was markedly inhibited in the 0.45 and 0.9 g/m^3^ groups (Figure 2A,E,F).

No obvious alterations in the total protein levels of AMPK, mTOR and ULK1 were observed after 1,2-DCE treatment. Nevertheless, p-AMPK (*p* = 0.0002, *p* < 0.0001) was upregulated in the 0.45 and 0.9 g/m^3^ groups, and p-ULK1 (*p* = 0.0005) was increased in the 0.9 g/m^3^ group. In contrast, p-mTOR expression was significantly suppressed in the 0.9 g/m^3^ group compared with that of the other treatment groups (*p* < 0.0001, Figure 2G–J).

### 3.3. 2-CE Exposure Induces Mitochondrial Damage and Apoptosis in PC12 Cells

**Figure 3:**
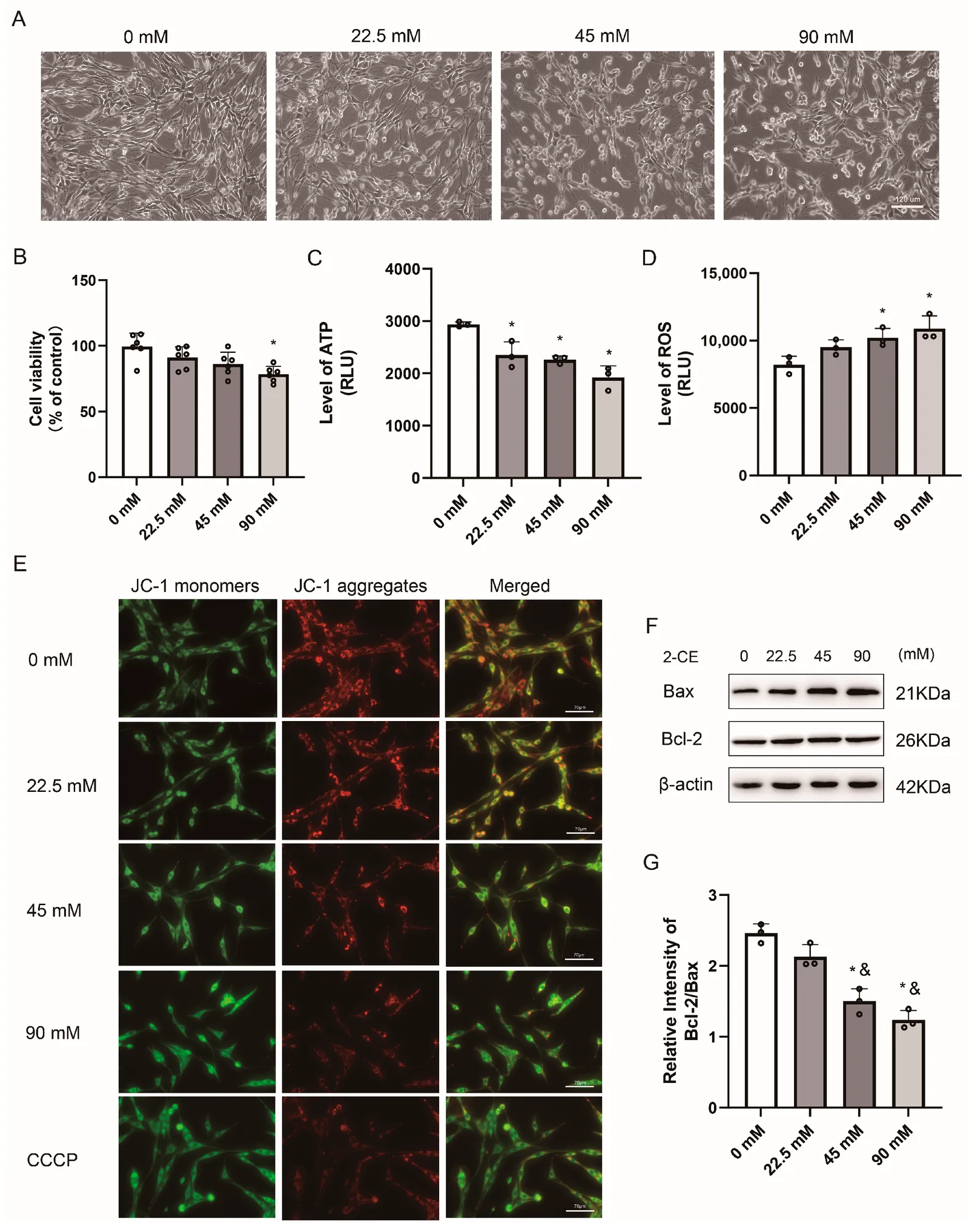
2-CE exposure induces mitochondrial damage and apoptosis in PC12 cells. (**A**) Cell morphology of PC12 cells. (**B**) Cell viability of PC12 cells. n = 6. (**C**) ATP levels in PC12 cells. n = 3. (**D**) ROS levels in PC12 cells. n = 3. (**E**) Changes in mitochondrial membrane potential in PC12 cells. CCCP was used as the positive control. (**F**) Representative western blot bands of BAX and Bcl-2. (**G**) Quantitative analysis of Bcl-2/BAX ratio. n = 3. *, vs. control; &, vs. 22.5 mM, *p* < 0.05.

With increasing 2-CE concentration, cell density and intercellular connections were gradually reduced (Figure 3A). Meanwhile, cell viability in the 90 mM group decreased to approximately 79%, significantly lower than that of the control group. Accordingly, 0, 22.5, 45 and 90 mM 2-CE were selected for subsequent experiments (Figure 3B).

As shown in Figure 3C, ATP levels were significantly decreased by 19%, 22% and 34% in the 22.5, 45 and 90 mM groups, respectively (*p* = 0.0134, *p* = 0.006, *p* = 0.0004). Meanwhile, ROS levels were elevated by 25% and 33% in the 45 and 90 mM groups (*p* = 0.0383, *p* = 0.0087, Figure 3D). Compared with the control group, red fluorescence intensity was markedly reduced in the 45 and 90 mM groups, whereas green fluorescence intensity increased progressively, indicating significant mitochondrial membrane potential depolarization (Figure 3E). In addition, the Bcl-2/Bax ratio was remarkably downregulated by 38% and 46% in the 45 and 90 mM groups, respectively (*p* = 0.0003, *p* < 0.0001, Figure 3F,G).

### 3.4. 2-CE Exposure Induces AMPK-mTOR-ULK1 Pathway Activation and Autophagic Flux Impairment in PC12 Cells

**Figure 4:**
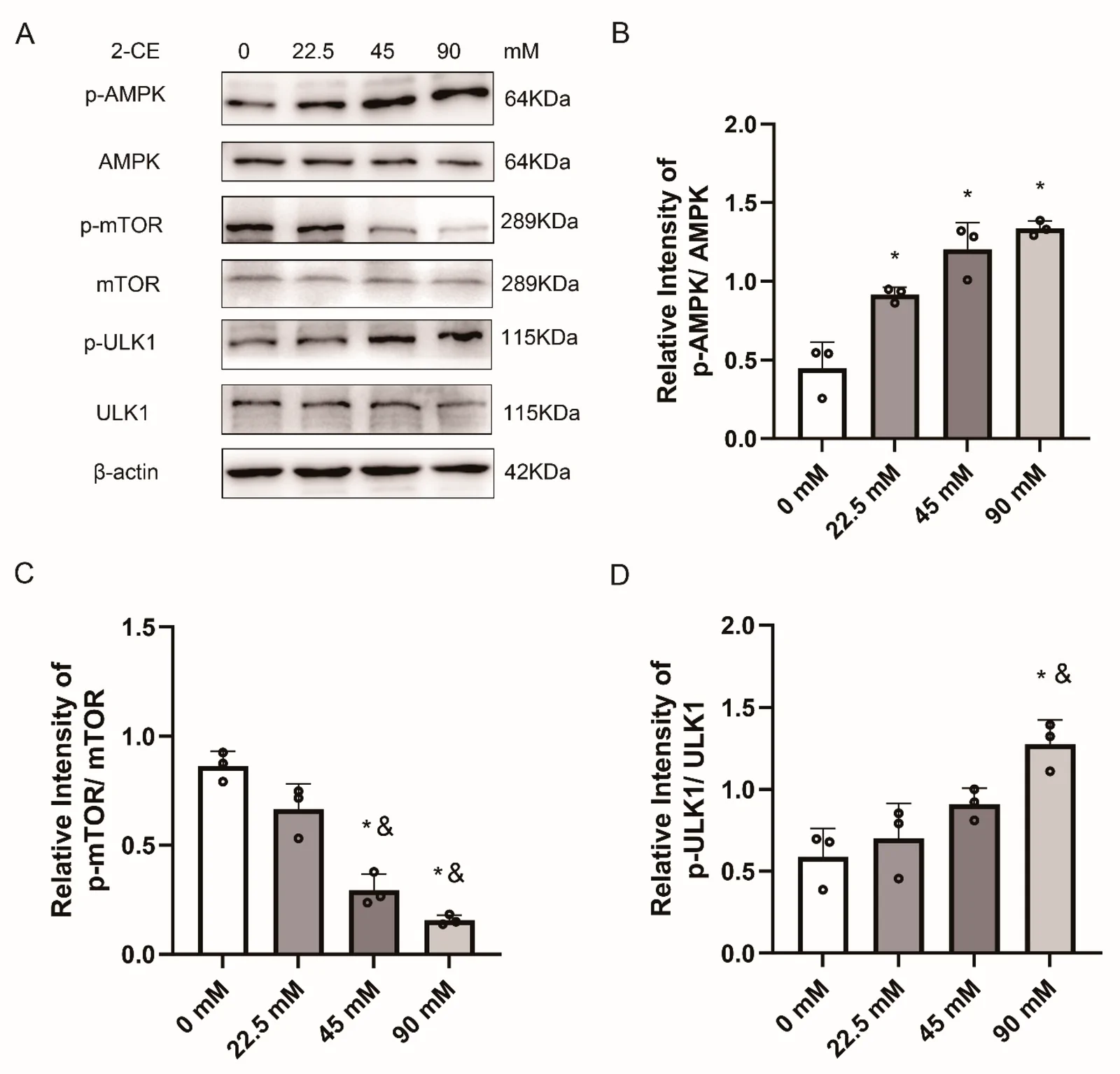
2-CE exposure induces AMPK-mTOR-ULK1 pathway activation in PC12 cells. (**A**) Representative western blot bands of p-AMPK, AMPK, p-mTOR, mTOR, p-ULK1 and ULK1. (**B**) Quantitative analysis of p-AMPK/AMPK ratio. (**C**) Quantitative analysis of p-mTOR/mTOR ratio. (**D**) Quantitative analysis of p-ULK1/ULK1 ratio. n = 3. *, vs. control; &, vs. 22.5 mM, *p* < 0.05.

Western blot analysis showed that the protein levels of p-AMPK (*p* < 0.0001) and p-ULK1 (*p* = 0.0038) were significantly upregulated in PC12 cells following 2-CE treatment, while p-mTOR expression was markedly suppressed in the 45 and 90 mM groups (*p* < 0.0001, Figure 4).

**Figure 5:**
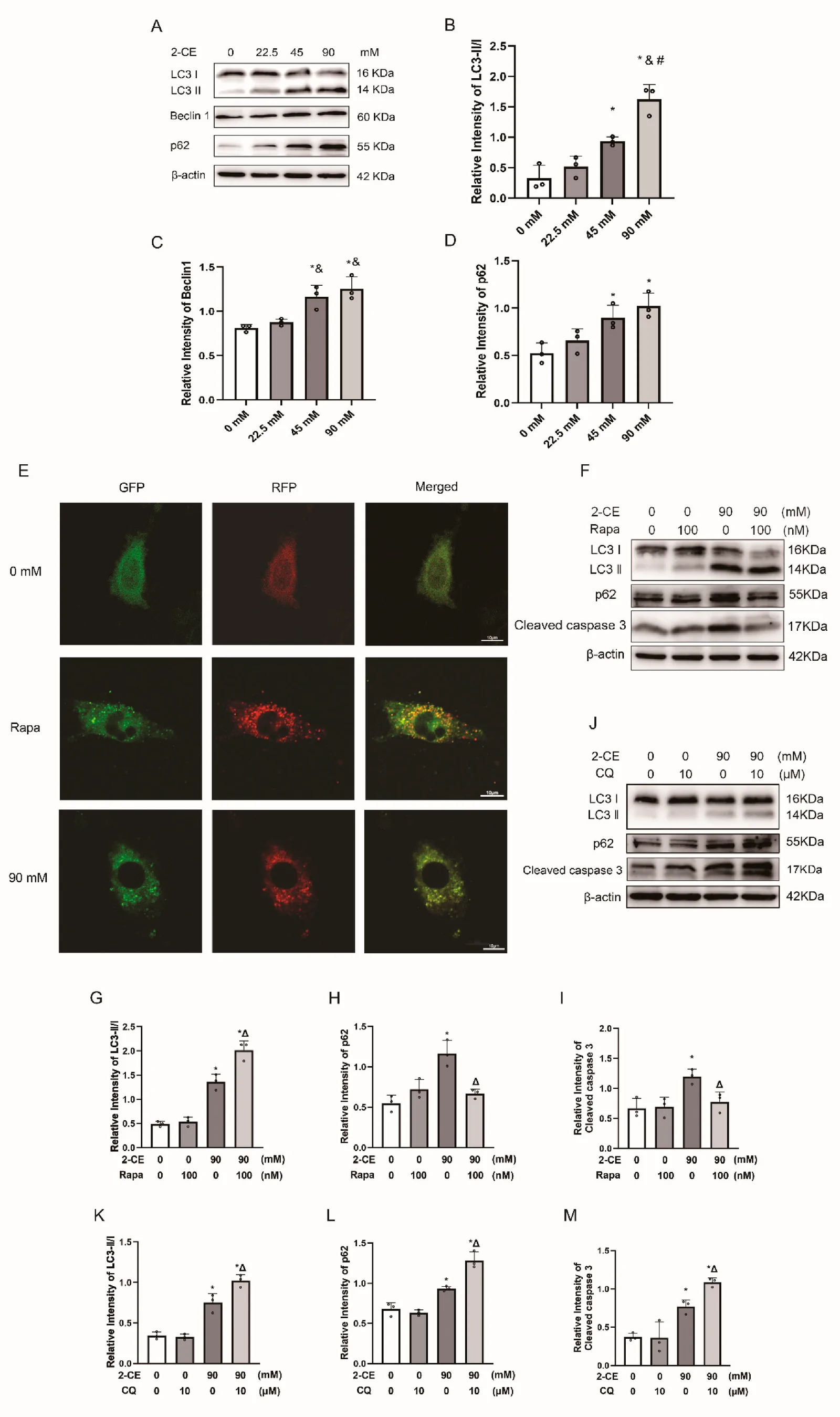
Rapa and CQ modulate 2-CE-induced autophagic flux and apoptosis in PC12 cells. (**A**) Representative western blot bands of LC3-II/I, Beclin 1 and p62. (**B**) Quantitative analysis of the LC3-II/I ratio. (**C**) Quantitative analysis of Beclin 1 expression. (**D**) Quantitative analysis of p62 expression. (**E**) Representative fluorescence images of autophagic flux. (**F**) Representative western blot bands of LC3-II/I, p62 and cleaved caspase 3 after Rapa intervention. (**G**) Quantitative analysis of the LC3-II/I ratio after Rapa treatment. (**H**) Quantitative analysis of p62 expression after Rapa treatment. (**I**) Quantitative analysis of cleaved caspase 3 after Rapa treatment. (**J**) Representative western blot bands of LC3-II/I, p62 and cleaved caspase 3 after CQ intervention. (**K**) Quantitative analysis of the LC3-II/I ratio after CQ treatment. (**L**) Quantitative analysis of p62 expression after CQ treatment. (**M**) Quantitative analysis of cleaved caspase 3 expression after CQ treatment. n = 3. *, vs. control; &, vs. 22.5 mM; #, vs. 45 mM; Δ, vs. 90 mM, *p* < 0.05.

Furthermore, the LC3-II/I ratio (*p* = 0.0164, *p* = 0.0001), Beclin 1 (*p* = 0.0097, *p* = 0.0025) and p62 (*p* = 0.0283, *p* = 0.0058) levels were markedly elevated in the 45 and 90 mM groups relative to those of the control group (Figure 5A–D). As shown in Figure 5E, the number of yellow puncta (autophagosomes) in the Rapa and 90 mM groups was significantly increased compared with that of the control group. However, compared with the Rapa group, the number of red puncta (autolysosomes) was significantly decreased in the 90 mM group.

Further pharmacological intervention experiments demonstrated that, in the 2-CE combined with Rapa group compared to the 2-CE-only group, Rapa reduced p62 expression by 42% (*p* = 0.0035, Figure 5H), decreased cleaved caspase-3 by 32% (*p* = 0.0138, Figure 5I), and increased the LC3-II/I ratio by 48% (*p* = 0.0017, Figure 5G). In contrast, CQ increased p62 expression by 37% (*p* = 0.0013, Figure 5L), the LC3-II/I ratio by 36% (*p* = 0.0082, Figure 5K), and cleaved caspase-3 by 41% (*p* = 0.0452, Figure 5M).

### 3.5. 3-MA Intervention Alleviates Autophagosome Accumulation and Reduces Apoptosis in PC12 Cells Induced by 2-CE Exposure

**Figure 6:**
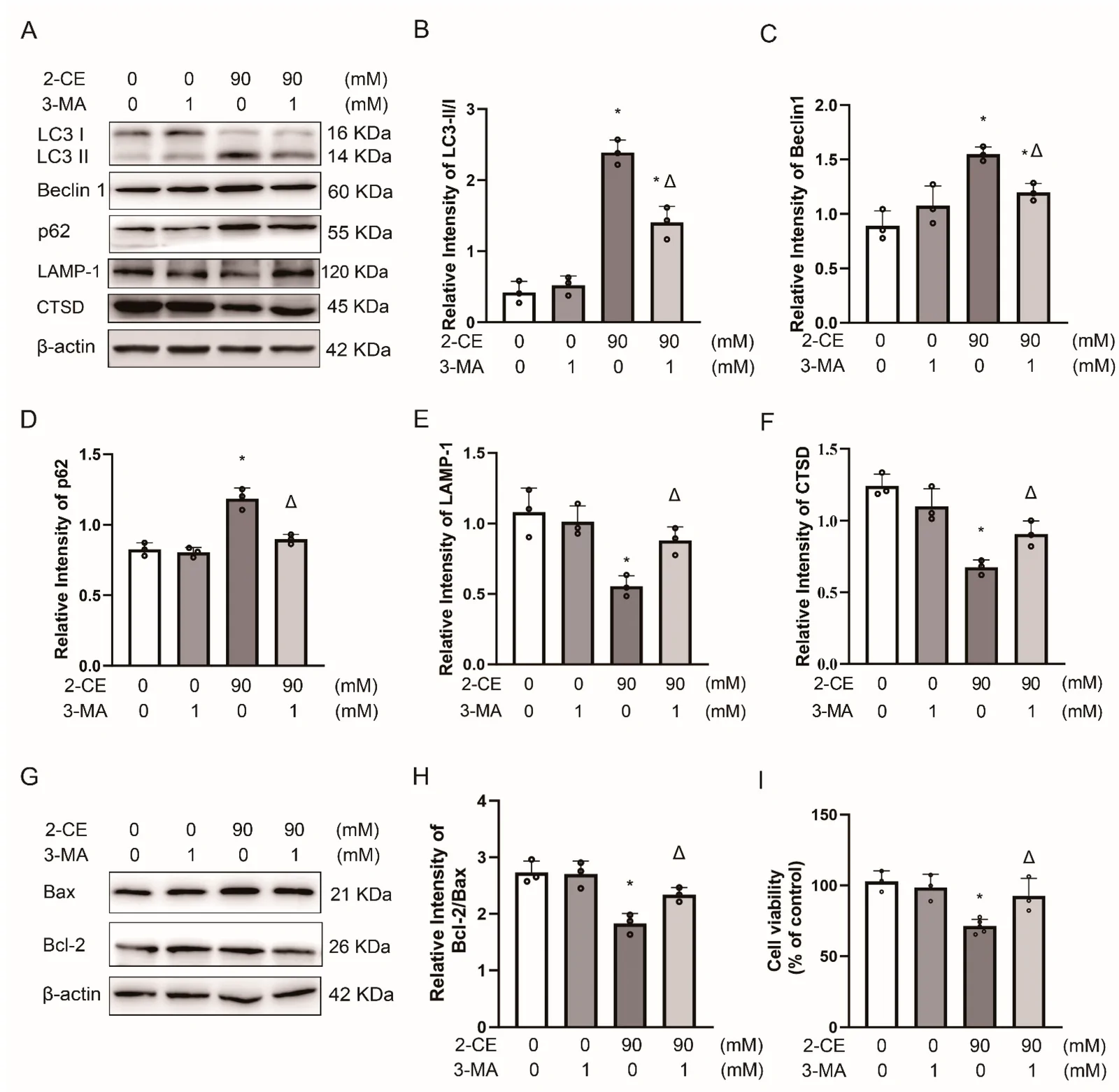
3-MA intervention alleviates autophagosome accumulation and reduces apoptosis in 2-CE-treated PC12 cells. (**A**) Representative western blot bands of LC3-II/I, Beclin 1, p62, LAMP1 and CTSD. (**B**) Quantitative analysis of the LC3-II/I ratio. (**C**) Quantitative analysis of Beclin 1 expression. (**D**) Quantitative analysis of p62 expression. (**E**) Quantitative analysis of LAMP1 expression. (**F**) Quantitative analysis of CTSD expression. (**G**) Representative western blot bands of BAX and Bcl-2. (**H**) Quantitative analysis of the Bcl-2/BAX ratio. (**I**) Cell viability after 3-MA treatment. n = 3. *, vs. control; Δ, vs. 90 mM, *p* < 0.05.

Western blot results revealed that, compared with the 2-CE alone group, 3-MA significantly reduced the LC3-II/I ratio (*p* = 0.0006), Beclin 1 (*p* = 0.0354) and p62 (*p* = 0.0005) levels by 41%, 22% and 24%, respectively (Figure 6A–D). By contrast, 3-MA treatment significantly reversed the inhibitory effect of 2-CE on LAMP1 (*p* = 0.0399) and CTSD (*p* = 0.0126) expression in PC12 cells (Figure 6E,F).

Meanwhile, the Bcl-2/Bax ratio in the 2-CE plus 3-MA group was significantly higher by 28% than that in the 2-CE alone group (*p* = 0.0424, Figure 6G,H). Furthermore, 3-MA treatment markedly attenuated the inhibitory effect of 2-CE on PC12 cell viability (*p* = 0.0227, Figure 6I).

### 3.6. Trehalose Treatment Restores Autophagic Flux and Reduces Apoptosis in PC12 Cells Induced by 2-CE Exposure

**Figure 7:**
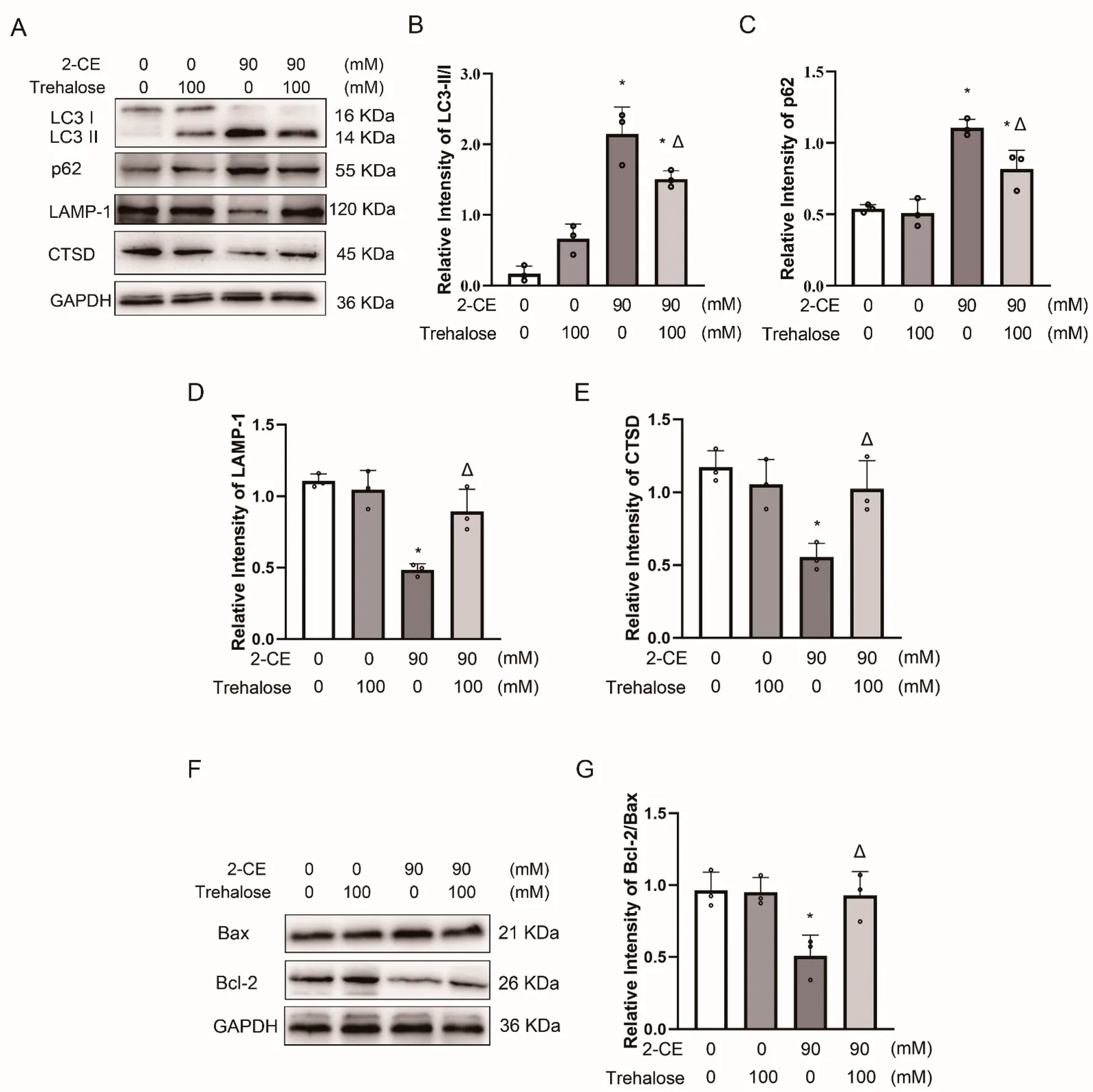
Trehalose treatment restores autophagic flux and reduces apoptosis in 2-CE-treated PC12 cells. (**A**) Representative western blot bands of LC3-II/I, p62, LAMP1 and CTSD. (**B**) Quantitative analysis of the LC3-II/I ratio. (**C**) Quantitative analysis of p62 expression. (**D**) Quantitative analysis of LAMP1 expression. (**E**) Quantitative analysis of CTSD expression. (**F**) Representative western blot bands of BAX and Bcl-2. (**G**) Quantitative analysis of the Bcl-2/BAX ratio. n = 3. *, vs. control; Δ, vs. 90 mM, *p* < 0.05.

The trehalose concentration was selected based on a previous study [11], and was confirmed to have no significant effect on cell viability in pilot experiments. Our results showed that trehalose significantly downregulated the LC3-II/I ratio (*p* = 0.04) by 30% and p62 (*p* = 0.0163) expression by 26%, while markedly upregulating LAMP1 (*p* = 0.0068) and CTSD (*p* = 0.0201) levels by 80% and 78%, respectively (Figure 7A–E). In addition, the Bcl-2/Bax ratio in trehalose-treated PC12 cells was significantly higher by 47% than that in the 2-CE alone group (*p* = 0.0229, Figure 7F,G).

### 3.7. AMPK-mTOR-ULK1 Signaling Mediates 2-CE-Induced Autophagy and Subsequent Apoptosis in PC12 Cells

**Figure 8:**
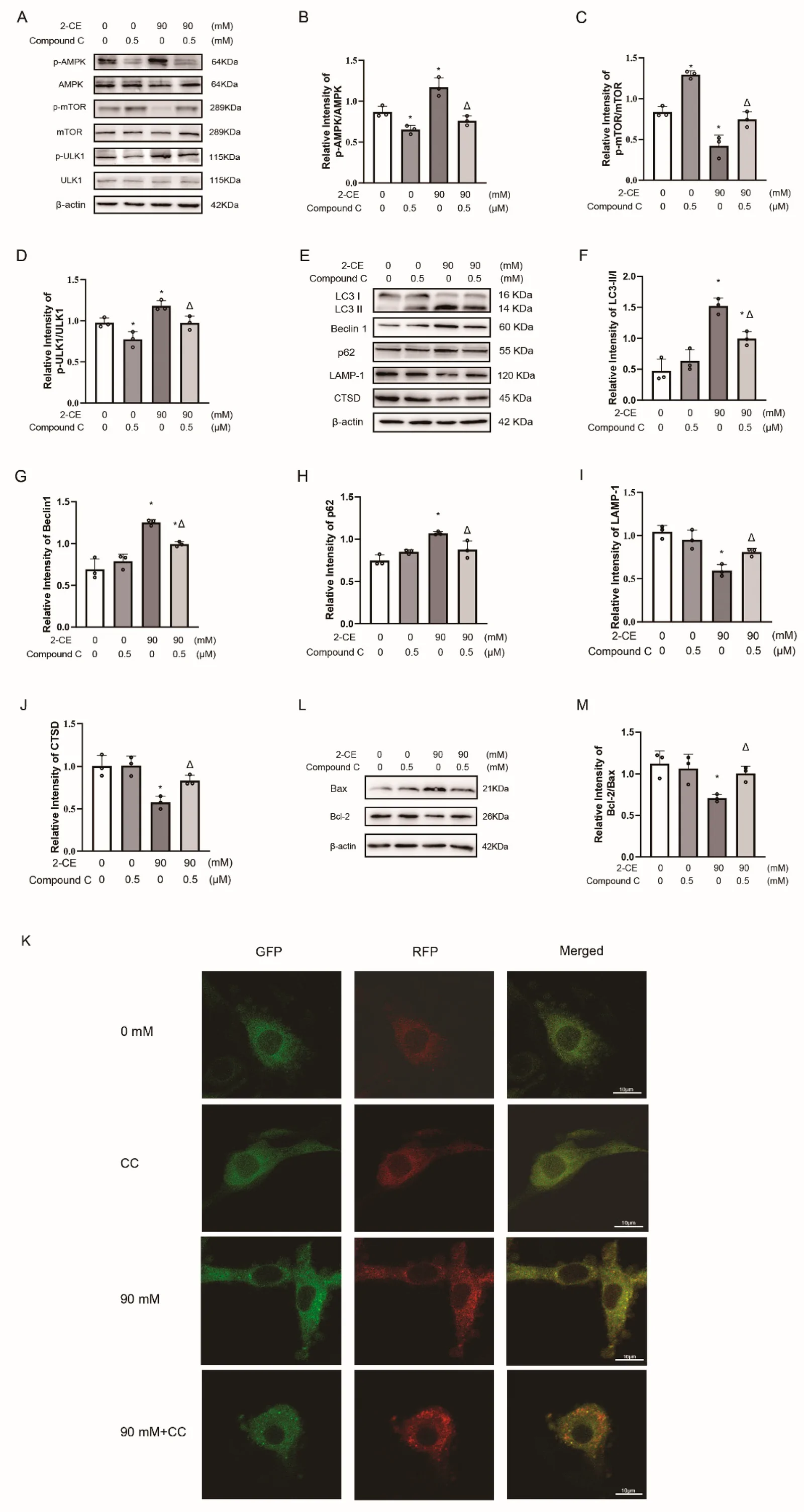
AMPK-mTOR-ULK1 signaling mediates 2-CE-induced autophagy and subsequent apoptosis in PC12 cells. (**A**) Representative western blot bands of p-AMPK, AMPK, p-mTOR, mTOR, p-ULK1 and ULK1. (**B**) Quantitative analysis of the p-AMPK/AMPK ratio. (**C**) Quantitative analysis of the p-mTOR/mTOR ratio. (**D**) Quantitative analysis of the p-ULK1/ULK1 ratio. (**E**) Representative western blot bands of LC3-II/I, Beclin 1, p62, LAMP1 and CTSD. (**F**) Quantitative analysis of the LC3-II/I ratio. (**G**) Quantitative analysis of Beclin 1 expression. (**H**) Quantitative analysis of p62 expression. (**I**) Quantitative analysis of LAMP1 expression. (**J**) Quantitative analysis of CTSD expression. (**K**) Representative fluorescence images of autophagic flux. (**L**) Representative western blot bands of BAX and Bcl-2. (**M**) Quantitative analysis of the Bcl-2/BAX ratio. n = 3. *, vs. control; Δ, vs. 90 mM, *p* < 0.05.

AMPK serves as a core upstream regulator of mTOR. To further clarify the interaction between autophagy and apoptosis, the AMPK-specific inhibitor CC was used for pharmacological intervention. Western blot results showed that CC effectively blocked the 2-CE-induced upregulation of p-AMPK (*p* = 0.0009) and p-ULK1 (*p* = 0.0417), while simultaneously increasing p-mTOR (*p* = 0.0102) expression (Figure 8A–D). In parallel, the LC3-II/I ratio (*p* = 0.0142), Beclin 1 (*p* = 0.0172) and p62 (*p* = 0.0227) levels were markedly downregulated in the 2-CE plus CC group relative to that of the 2-CE alone group (Figure 8E–H). Moreover, CC intervention obviously reversed the suppressive effect of 2-CE on the expression of LAMP1 and CTSD (*p* = 0.0171, *p* = 0.0366, Figure 8E,I,J). In the RFP-GFP-LC3B fluorescence assay, green fluorescence was partially quenched after CC pretreatment, and the number of red puncta (autolysosomes) was significantly higher compared with that of the single 90 mM 2-CE group (Figure 8K). These findings indicated that CC treatment restrained 2-CE-triggered excessive autophagosome accumulation. In addition, the Bcl-2/Bax ratio was distinctly increased in the CC-pretreated group compared with that of the 2-CE alone group (*p* = 0.0172, Figure 8L,M).

## 4. Discussion

Our previous research indicates that, contrary to the subacute poisoning typically linked to high-dose 1,2-DCE inhalation [2], long-term exposure to concentrations below 1.0 g/m^3^ significantly impairs learning and memory in mice [5,6]. To elucidate this neurotoxic mechanism, this study employed a 1,2-DCE concentration range of 0.225–0.9 g/m^3^. Consistent with our prior findings, behavioral assessments revealed that mice exposed to 0.45 and 0.9 g/m^3^ 1,2-DCE exhibited significant deficits in learning and memory performance.

Neuronal loss is a primary mechanism of 1,2-DCE-induced neurotoxicity [16,19]. We observed hippocampal CA1 neuronal ultrastructural damage, including mitochondrial swelling and cristae loss. Consistently, 2-CE, the main neurotoxic metabolite of 1,2-DCE, recapitulated these defects in PC12 cells, with reduced MMP and elevated ROS. Notably, 0.225 g/m^3^ of 1,2-DCE in vivo or 22.5 mM of 2-CE in vitro significantly reduced ATP production, indicating that neuronal energy metabolism is highly sensitive to 1,2-DCE. As a highly metabolically active organ, the brain relies entirely on mitochondrial function to meet neuronal energy demands [20]. Mitochondria are exceptionally sensitive to environmental toxins, even at current safety limits [21], making them critical targets for exogenous chemicals [22]. Mitochondrial dysfunction is a common pathological basis underlying many human diseases [23]. Our data showed that long-term exposure to low doses of 1,2-DCE impaired the energy metabolic function of neuronal mitochondria, indicating that mitochondrial dysfunction is a key early target of 1,2-DCE neurotoxicity.

Both autophagy and apoptosis are tightly associated with mitochondrial dysfunction [24]. In the present study, enhanced apoptosis and autophagy initiation were observed in the hippocampus of 1,2-DCE-exposed mice with cognitive deficits, as evidenced by a decreased Bcl-2/Bax ratio and upregulated Beclin-1 protein expression.

As the central cellular energy sensor, AMPK was found to be activated in the hippocampus of mice following 1,2-DCE exposure in the present study, suggesting that AMPK activation may represent a downstream event triggered by 1,2-DCE-induced mitochondrial dysfunction. As a critical upstream regulator of mTOR, AMPK exerts a critical role in autophagy initiation [25,26,27]. We observed that 1,2-DCE exposure elevated the phosphorylation levels of AMPK and ULK1 in the mouse hippocampus, whereas the phosphorylation of mTOR was suppressed. Taken together, these findings indicate that autophagy activation in the hippocampus after 1,2-DCE exposure is closely associated with activation of the AMPK-mTOR-ULK1 signaling pathway. To further validate this hypothesis, we treated PC12 cells with the AMPK-specific inhibitor CC in vitro. The results suggest that CC treatment not only markedly inhibited the AMPK-mTOR-ULK1 signaling cascade, but also attenuated 2-CE-induced autophagy activation in PC12 cells.

Accumulating evidence has established extensive crosstalk between autophagy and apoptosis. Autophagy generally precedes apoptosis and confers cytoprotection against apoptosis through multiple mechanisms, including the clearance of damaged mitochondria to prevent cytochrome c release, p62-mediated antioxidant defense via activation of the Nrf2 pathway, the interaction between Beclin-1 and Bcl-2 family proteins, and p53-mediated regulation [28,29]. However, when cellular stress exceeds a certain threshold, it triggers programmed cell death, such as apoptosis. Numerous studies have confirmed the complex regulatory relationship between autophagy and apoptosis. Autophagy may promote cell survival and avoid apoptosis, eventually lead to cell death, act together with apoptosis, or exist independently when apoptosis fails [10]. A recent review by Gupta et al. [29] noted that autophagy exerts a dual role in neuronal cells through the activation or inhibition of apoptosis signaling.

In the present study, enhanced autophagy initiation was found to be accompanied by enhanced apoptosis in response to 2-CE exposure in PC12 cells. Inhibition of autophagy by 3-MA and CC, respectively, resulted in decreased apoptosis, indicating that autophagy is involved in the apoptotic process induced by 2-CE in PC12 cells. Notably, CQ-mediated inhibition of autophagolysosomal degradation led to a significant increase in both the LC3-II/I ratio and p62 accumulation [30]. In contrast, Rapa, which induces autophagy, significantly increased the LC3-II/I ratio and decreased p62 accumulation [31]. These findings suggest that 2-CE disrupts the homeostasis of autophagic flux.

In this study, the upregulation of p62 expression in vivo, combined with the colocalization results of RFP-GFP-LC3B in PC12 cells exposed to 2-CE in vitro, collectively indicated that 1,2-DCE exposure induced autophagic flux impairment. This conclusion was further confirmed by interventions with Rapa and CQ. Notably, after 6 h and 12 h of treatment with 90 mM 2-CE, p62 levels and the LC3-II/I ratio remained relatively low but showed a marked elevation at 24 h and 48 h (Figure S1), indicating that 2-CE-induced autophagic flux impairment emerged only upon prolonged exposure. To date, only a single study has reported impaired autophagic flux in midbrain optic tectum neurons of zebrafish following 1,2-DCE exposure [32].

Accumulating evidence has indicated that LAMP1 and CTSD play critical roles in the autophagosome-lysosome degradation process [33,34]. Therefore, to further elucidate the mechanism underlying 1,2-DCE-induced autophagic flux impairment, we examined the protein levels of these two lysosomal markers. Our data showed that LAMP1 and CTSD expression was significantly reduced in the hippocampus of 1,2-DCE-exposed mice. Subsequently, trehalose [35], a natural disaccharide that enhances autophagy-lysosomal system function, was used for in vitro intervention. We found that trehalose treatment significantly restored the protein levels of LAMP1 and CTSD, while downregulating the LC3-II/I ratio and p62 levels in PC12 cells. These findings collectively indicate that impaired autophagosome-lysosome fusion or reduced lysosomal number and function ultimately caused autophagic flux impairment in 1,2-DCE-exposed mice.

Increasing studies have demonstrated that autophagic flux impairment leads to autophagosome accumulation and further aggravates the toxic effects of environmental toxicants on neurons, ultimately inducing apoptosis [36]. In the present study, trehalose treatment restored functional autophagic flux and concomitantly attenuated apoptosis in PC12 cells. These findings further indicate that defective autophagosome-lysosome fusion or lysosomal dysfunction constitutes a critical downstream mechanism underlying 1,2-DCE-induced autophagic flux impairment and subsequent neuronal apoptosis. This finding was further corroborated by in vitro experiments using CQ and Rapa.

Based on these findings, we hypothesized that reducing autophagosome formation at the initiation stage would alleviate the accumulated pressure of autophagic substrates caused by autophagic flux impairment, thereby partially restoring lysosomal function and reducing apoptosis. To test this hypothesis, we employed 3-MA, an inhibitor of class III PI3K that blocks autophagy initiation. 3-MA treatment significantly reduced the LC3-II/I ratio, Beclin-1, and p62 protein levels, while partially restoring LAMP1 and CTSD expression and ultimately attenuating apoptosis in 2-CE-exposed PC12 cells. Some research findings are similar to ours [37,38]. Notably, 3-MA inhibited autophagy by targeting the AMPK-mTOR signaling axis downstream. To further investigate whether AMPK acts as an important upstream regulator, we used its inhibitor CC. Similar results were also obtained after CC intervention. These results suggest that inhibiting autophagy initiation from the upstream can partially alleviate autophagic flux impairment and its downstream apoptotic consequences. The underlying mechanism likely involves autophagic stress, defined as a condition in which the rate of autophagosome formation exceeds that of lysosomal degradation [39,40]. In our study, 1,2-DCE concurrently activated autophagy initiation via the AMPK-mTOR-ULK1 signaling axis while impairing lysosomal function. This dual action disrupted autophagic flux, resulting in sustained accumulation of autophagosomes and progressive lysosomal insufficiency. Consequently, the accumulating autophagic cargo overloads the compromised lysosomal system, exacerbating lysosomal dysfunction and triggering apoptotic cell death. A recent study reported that AMPK activation rapidly downregulates Rab5, Rab7, and LAMP1 through translation inhibition and proteasome-dependent degradation, suggesting a post-translational pathway by which AMPK may directly suppress endo-lysosomal protein expression [41]. Previous studies have shown that 1,2-DCE and its metabolites induce apoptosis through multiple signaling pathways, including the CREM/CREB pathway, ERK1/2 pathway, and non-coding RNA regulation [16,19,42,43,44]. 1,2-DCE concurrently activates autophagy initiation via the AMPK-mTOR-ULK1 signaling axis while impairing lysosomal function, leading to autophagic flux impairment and apoptotic cell death. Consistently, several studies have reported that autophagy activated via the AMPK-mTOR-ULK1 pathway promotes apoptosis [15,45,46]. By contrast, other studies have demonstrated that AMPK-mTOR-ULK1-triggered autophagy inhibits apoptosis [47,48]. Collectively, the effect of AMPK-mTOR-ULK1-mediated autophagy on apoptosis appears to be context-dependent, and the exact underlying mechanisms warrant further investigation.

This study has several limitations. First, in vivo experiments were performed only in female mice, because the majority of 1,2-DCE workers in China are female. Of course, this limits the generalization of the conclusions to male mice and prevents the evaluation of sex-dependent effects. The relatively small sample size may reduce statistical power. Second, neuronal loss or apoptosis in brain tissue was not quantitatively assessed using established methods such as TUNEL staining. Additionally, autophagic flux was evaluated without dynamic detection approaches, and lysosomal function was not directly measured. Furthermore, the study did not employ in vivo genetic or pharmacological interventions, which somewhat limits the establishment of causal relationships. Regarding in vitro experiments, PC12 cells were used as a substitute model for hippocampal neurons, which may not fully recapitulate the signaling characteristics and vulnerability of primary neurons. The concentration of 2-CE used in vitro was relatively high, and its relevance to chronic low-dose occupational exposure remains unclear. Moreover, pharmacological modulators exhibit off-target effects and limited specificity. Despite these limitations, convergent evidence from both in vivo and in vitro studies provides preliminary support for the proposed mechanistic model. Future research should incorporate genetic models, dynamic autophagy flux measurements, and direct assessments of lysosomal function to validate and extend these findings.

## 5. Conclusions

Overall, our findings indicate for the first time that prolonged 1,2-DCE exposure induces mitochondrial dysfunction and ATP depletion in neurons and activates autophagy initiation via AMPK-mTOR-ULK1 signaling. The concomitant lysosomal damage leads to impaired autophagic flux, and this combined effect of activation of autophagy initiation and impaired autophagic degradation further promotes neuronal apoptosis. These findings provide a potential novel therapeutic target for the prevention and treatment of 1,2-DCE-induced cognitive impairment.

## Data Availability

The original contributions presented in the study are included in the article/Supplementary Materials, further inquiries can be directed to the corresponding authors.

## Supplementary Materials

The following supporting information can be downloaded at: https://www.mdpi.com/article/10.3390/biology15181580/s1, Figure S1: Prolonged 2-CE exposure triggers autophagic flux impairment in PC12 cells. (A) Representative western blot bands of LC3-II/I and p62. (B) Quantitative analysis of the LC3-II/I ratio. (C) Quantitative analysis of p62 expression. n = 3. *, vs. 6 h group; &, vs. 12 h group, *p* < 0.05. File S1: Original Western blot images for Figure 1, Figure 2, Figure 3, Figure 4, Figure 5, Figure 6, Figure 7 and Figure 8.
