# Supplementary material for: 1,2-Dichloroethane Induces Neuronal Apoptosis Through AMPK-mTOR-ULK1 Pathway Activation and Autophagic Flux Impairment
Source: Biology (Basel). 2026 Sep 9;15(18):1580. doi: 10.3390/biology15181580 (PMC13604869; doi:10.3390/biology15181580)
Supplement: Supplementary file 1 [file biology-15-01580-s001.zip › Supplementary materials.pdf]

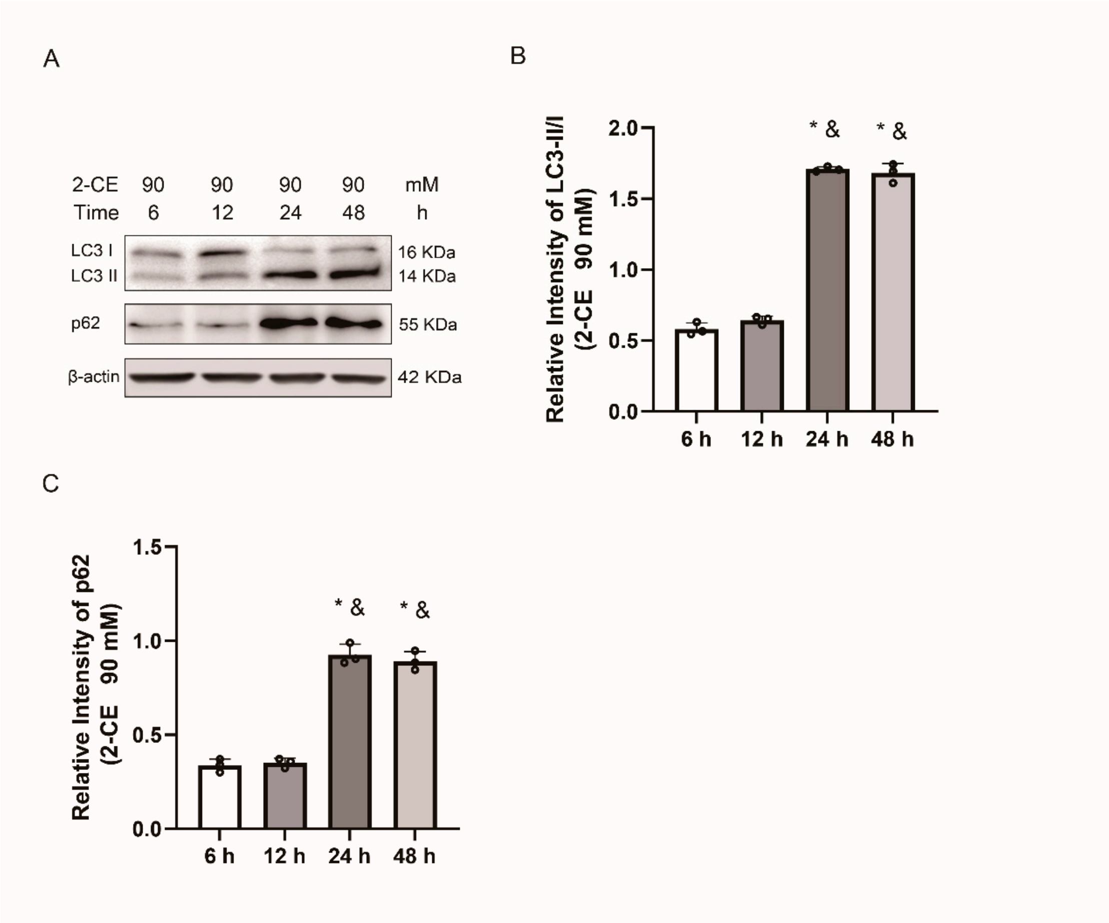

Figure S1. Prolonged 2-CE exposure triggers autophagic flux impairment in PC12 cells. (A) Representative western blot bands of LC3-II/I and p62. (B) Quantitative analysis of the LC3-II/I ratio. (C) Quantitative analysis of p62 expression. n=3. *, vs 6 h group; &, vs 12 h group, $P < 0.05$.
